# Long non-coding RNA NEAT1 overexpression is associated with poor prognosis in cancer patients: a systematic review and meta-analysis

**DOI:** 10.18632/oncotarget.13737

**Published:** 2016-12-01

**Authors:** Chao Yang, Zhuo Li, Yajun Li, Rui Xu, Yongfeng Wang, Yu Tian, Wei Chen

**Affiliations:** ^1^ Department of Clinical Laboratory, The First Affiliated Hospital of Xi'an Medical University, Xi'an City, Shaanxi Province, China; ^2^ Department of Neurology, The First Affiliated Hospital of Xi'an Medical University, Xi'an City, Shaanxi Province, China; ^3^ Department of Clinical Research, The First Affiliated Hospital of Xi'an Medical University, Xi'an City, Shaanxi Province, China; ^4^ Department of Clinical Laboratory, The First Affiliated Hospital of Xi'an Jiaotong University, Xi'an City, Shaanxi Province, China

**Keywords:** lncRNA, NEAT1, prognosis, cancer, meta-analysis

## Abstract

**Objectives:**

Long non-coding RNAs (lncRNAs) are playing important roles in cancer progression and metastasis. Recent studies have demonstrated that the lncRNA, nuclear paraspeckle assembly transcript 1 (NEAT1), was aberrantly up-regulated in various types of cancers and was reported to be associated with unfavorable prognosis in cancer patients. This study examined the relationship between NEAT1 and relevant clinical outcomes.

**Results:**

A total of 1354 patients from 11 eligible studies were included in the meta-analysis. The results showed that high expression level of NEAT1 was significantly associated with shorter overall survival in cancer patients (hazard ratio (HR) = 1.53, 95% confidence interval (CI) = 1.36–1.71); in the subgroup analysis, the positive association was also found in patients with hepato-gastroenterol cancers (HR = 1.79, 95% CI = 1.48–2.16), non-small cell lung cancer (HR = 1.35, 95% CI = 1.04–1.76), ovarian cancer (HR = 1.41, 95% CI = 1.11–1.79) and other types of cancers (HR = 1.42, 95% CI = 1.11–1.81). The clinicopathological parameters analysis further showed that increased expression level of NEAT1 was positively correlated with larger tumor size (odds ratio (OR) = 1.74, 95% CI = 1.26–2.41), lymph node metastasis (OR = 2.29, 95% CI = 1.71–3.06), advanced TNM stage (OR = 3.60, 95% CI = 2.27–5.72), poor tumor differentiation (OR = 2.16, 95% CI = 1.58–2.93), distant metastasis (OR = 3.51, 95% CI = 1.75–7.01), and invasion depth (OR = 1.94, 95% CI = 1.36–2.75).

**Materials and Methods:**

A comprehensive search was performed in Pubmed, Embase, Web of Science and CNKI databases, and eligible studies were included based on defined exclusion and inclusion criteria to perform meta-analysis.

**Conclusions:**

The meta-analysis results from present study suggested that increased expression level of NEAT1 was associated with unfavorable prognosis and may serve as a predictive factor for clinicopathological features in various cancers.

## INTRODUCTION

Cancer is becoming a major public health problem and is one of the main causes of morbidity and mortality worldwide [1]. The overall cancer-related death rates were still expected to rise in the future due to increased number of newly-diagnosed cases and insufficient understanding of the molecular mechanisms underlying cancer development [2]. In addition, the 5-year survival rate is still very low in many types of human cancers. Therefore, it is necessary for us to identify new potential biomarkers for early diagnosis and prognosis, and novel potential therapeutic target for the treatment of cancers.

Long non-coding RNAs (lncRNAs) are transcribed RNA molecules with more than 200 nucleotides and can not code proteins [3]. Many studies have demonstrated the diverse cellular functions of lncRNAs including cell proliferation, cell differentiation, cell apoptosis and carcinogenesis [4]. In the last decade, numerous studies have reported the dysregulation of lncRNAs in cancer, and the dysregulation of lncRNAs was found to contribute to cancer progression and metastasis. The lncRNAs such as H19 [5], Metastasis Associated Lung Adenocarcinoma Transcript 1 [6], HOX transcript antisense intergenic RNA [7], urothelial cancer associated 1 [8], antisense non-coding RNA in the INK4 locus [9] and PVT1 [10] were found to be novel promising biomarkers to predict a poor prognosis and lymph node metastasis in human cancers . Recently, the lncRNAs, nuclear paraspeckle assembly transcript 1 (NEAT1), was reported to have a role in cancer prognosis and chemo-/radio-sensitivity in a substantial number of studies. NEAT1 was found to be a diagnostic and prognostic biomarker in colorectal cancer [10], and Li et al., 2015 further showed that NEAT1 up-regulation is associated with tumor recurrence and unfavorable prognosis [11]. Study also showed that NEAT1 was identified as a critical modulator of prostate cancer by interacting with oestrogen receptor alpha [12]. In addition, NEAT1 enhances non-small cell lung cancer (NSCLC) via regulation of miR-377-3p-E2F3 pathway [13]. Fu et al., 2016 also found that NEAT1 was an unfavorable prognostic factor and promotes migration and invasion in gastric cancer [14].

Up to date, no meta-analysis has been performed to examine the relationship between NEAT1 and the relevant clinical outcomes. In the present study, relevant publications were collected to investigate whether the increased expression of NEAT1 could be served as a potential biomarker for prognosis in cancer patients.

## RESULTS

### Study characteristics

**Figure 1:**
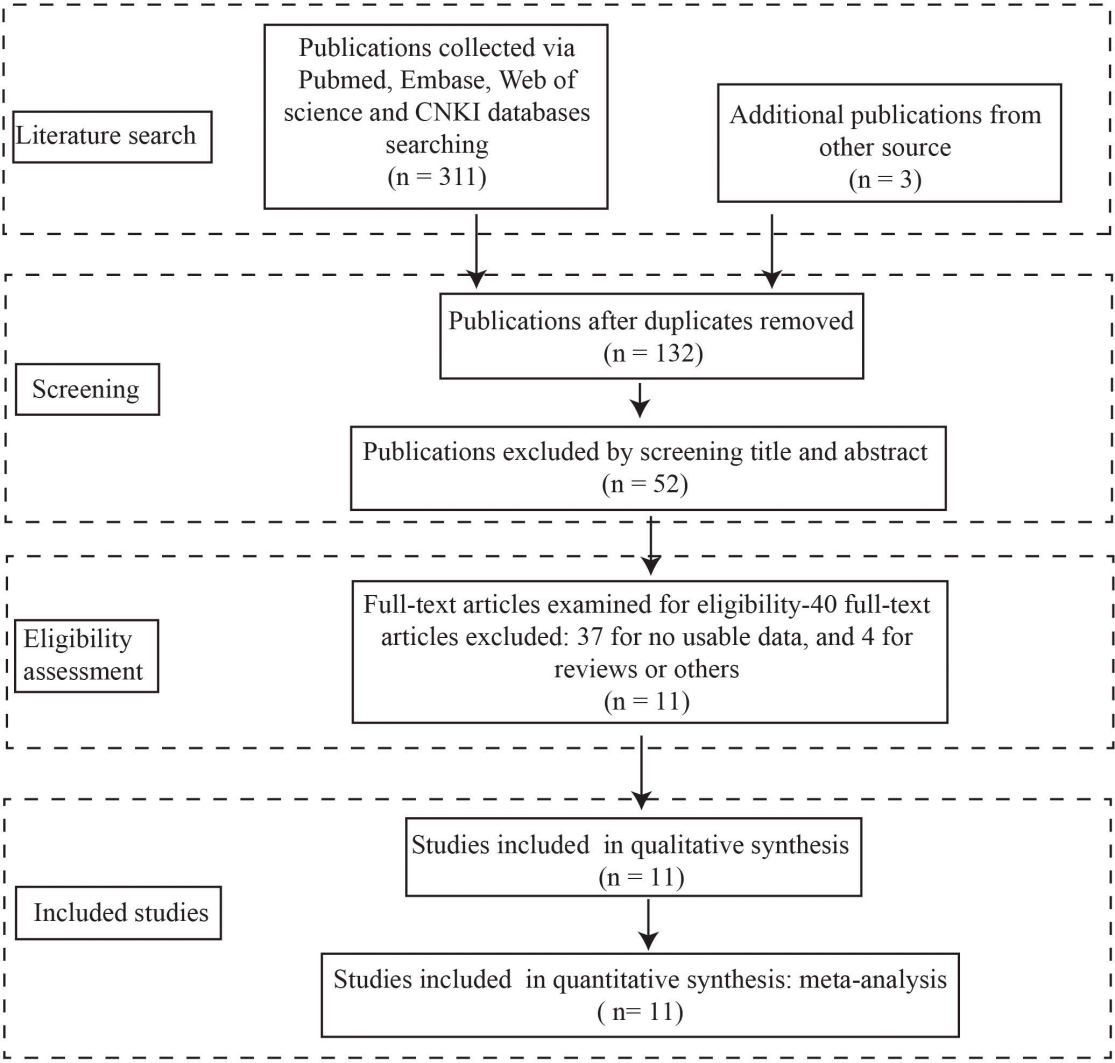
Stepwise procedures for searching databases and selecting eligible studies

The detailed procedures of literature retrieval were shown in Figure 1. A total of 11 studies were finally identified. The total number of patients included in the present meta-analysis was 1354, and the patient sample size ranges from 71 to 239 with a mean value of 96.0. Ten included studies were conducted in China, and one study was conducted in foreign countries. There are 8 type of cancers in the included studies, with two studies for ovarian cancer, two studies for colorectal cancer, two studies for NSCLC, one study for glioma, one study for nasopharyngeal carcinoma, one study for esophageal squamous cell carcinoma (ESCC), one study for hepatocellular carcinoma (HCC). All the clinical specimens were preserved before RNA extraction, and main information of the included studies were shown in Table 1.

**Table 1:** Summary of all included eligible studies

First Author	Year	Cancer type	Total number	Tumor stage	Follow-up (months)	Adjuvant therapy before surgery	Criterion of high expression	Detection method	Outcome measures	Multivariate analysis
Chen ZJ [28]	2016	Ovarian cancer	149	53/96(I-II/III-IV)	Over 60	None	Median expression	qRT-PCR	OS	Yes
Fu JW [14]	2016	Gastric cancer	140	63/77(I-II/III-IV)	Over 60	NR	Median expression	qRT-PCR	OS	Yes
Sun C [13]	2016	NSCLC	96	28/68 (I-II/III/IV)	Over 40	None	NR	qRT-PCR	OS	No
Adriaens C [19]	2016	Ovarian cancer	58	NR	Over 60	Yes	NR	qRT-PCR	OS, PFS	No
Guo S [29]	2016	HCC	95	22/73(I-II/III-IV)	Over 60	NR	Median expression	qRT-PCR	OS	No
Li Y [11]	2015	Colorectal cancer	239	92/147(I-II/III-IV)	Over 60	None	Fold change	qRT-PCR	OS, DFS	Yes
He C [30]	2015	Glioma	94	23/71 (I-II/III-IV)	Over 50	None	Median expression	qRT-PCR	OS	Yes
Pan LJ [31]	2015	NSCLC	125	54/71(I-II/III-IV)	Over 40	NR	Mean expression	qRT-PCR	OS	No
Chen X [32]	2015	ESCC	96	35/61 (I-II/III-IV)	Over 60	None	Youden index	qRT-PCR	OS	Yes
Wu Y [33]	2015	Colorectal cancer	191	26/165 (I-II/III-IV)	Over 60	None	Mean expression	qRT-PCR	OS	Yes
Lu Y [34]	2015	Nasopharyngeal carcinoma	71	36/35(I-II/III-IV)	Over 40	NR	NR	qRT-PCR	OS	No

Abbreviations: DFS = disease-free survival; ESCC = esophageal squamous cell carcinoma; HCC = hepatocellular carcinoma; NR = not reported; NSCLC = non-small cell lung cancer; OS = overall survival.

### The association between NEAT1 expression levels and overall survival (OS)

**Figure 2:**
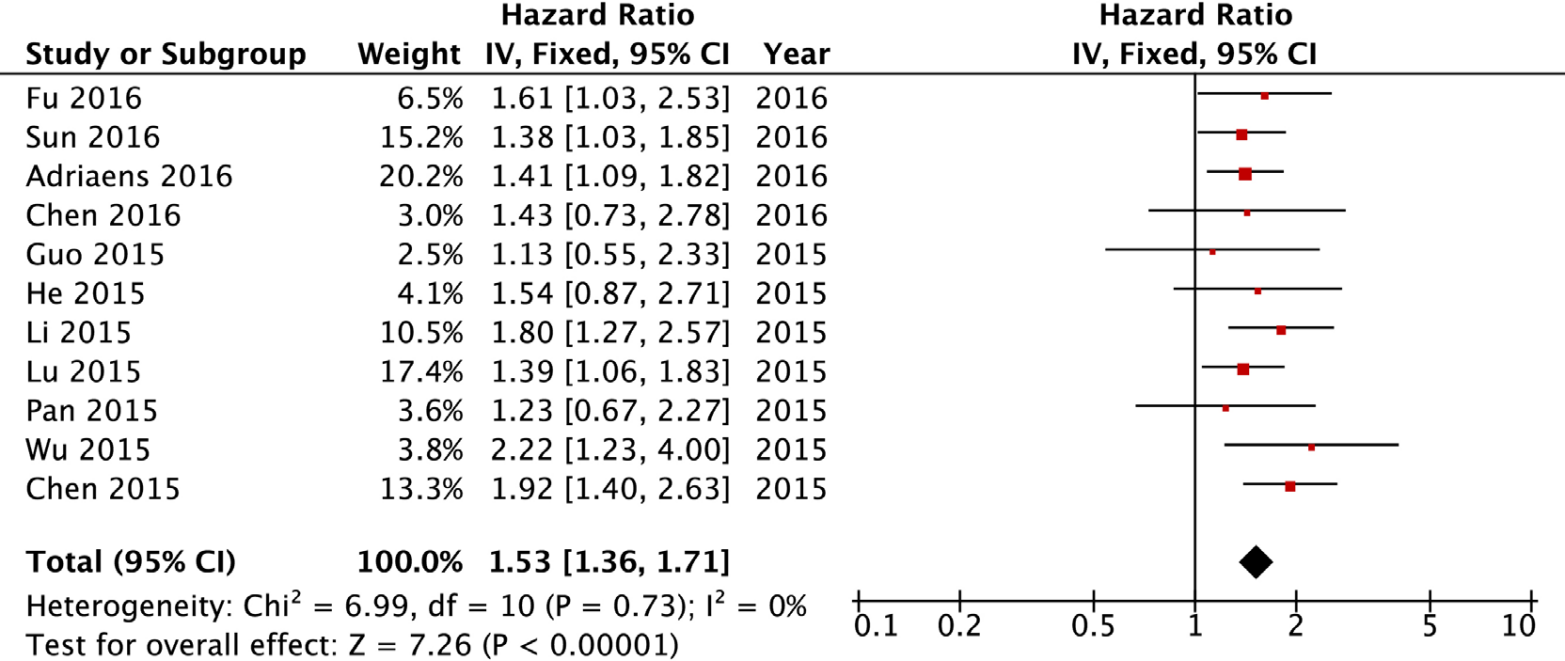
Forest plot of HRs for the association between high NEAT1 expression and OS in cancer patients

We first analyzed the association between NEAT1 expression levels and OS in the 11 included studies. The fixed-effects model was applied to estimate the pooled hazard ratios (HRs) and the respective 95% confidence interval (CI). As show in Figure 2, there was no heterogeneity across these included studies (P_h_ = 0.73, I^2^ = 0%). The HR of the high NEAT1 expression level group versus the low NEAT1 expression level group was 1.53 (95% CI = 1.36–1.71, *P <* 0.001, Figure 2). The results suggest that there was significant difference in the OS between high NEAT1 expression level group and low NEAT1 expression level group. A significantly shorter OS was shown in the patients with high NEAT1 expression level than that with low NEAT1 expression level. Thus, it is implied that the increased expression level of NEAT1 was associated with poor OS.

**Figure 3:**
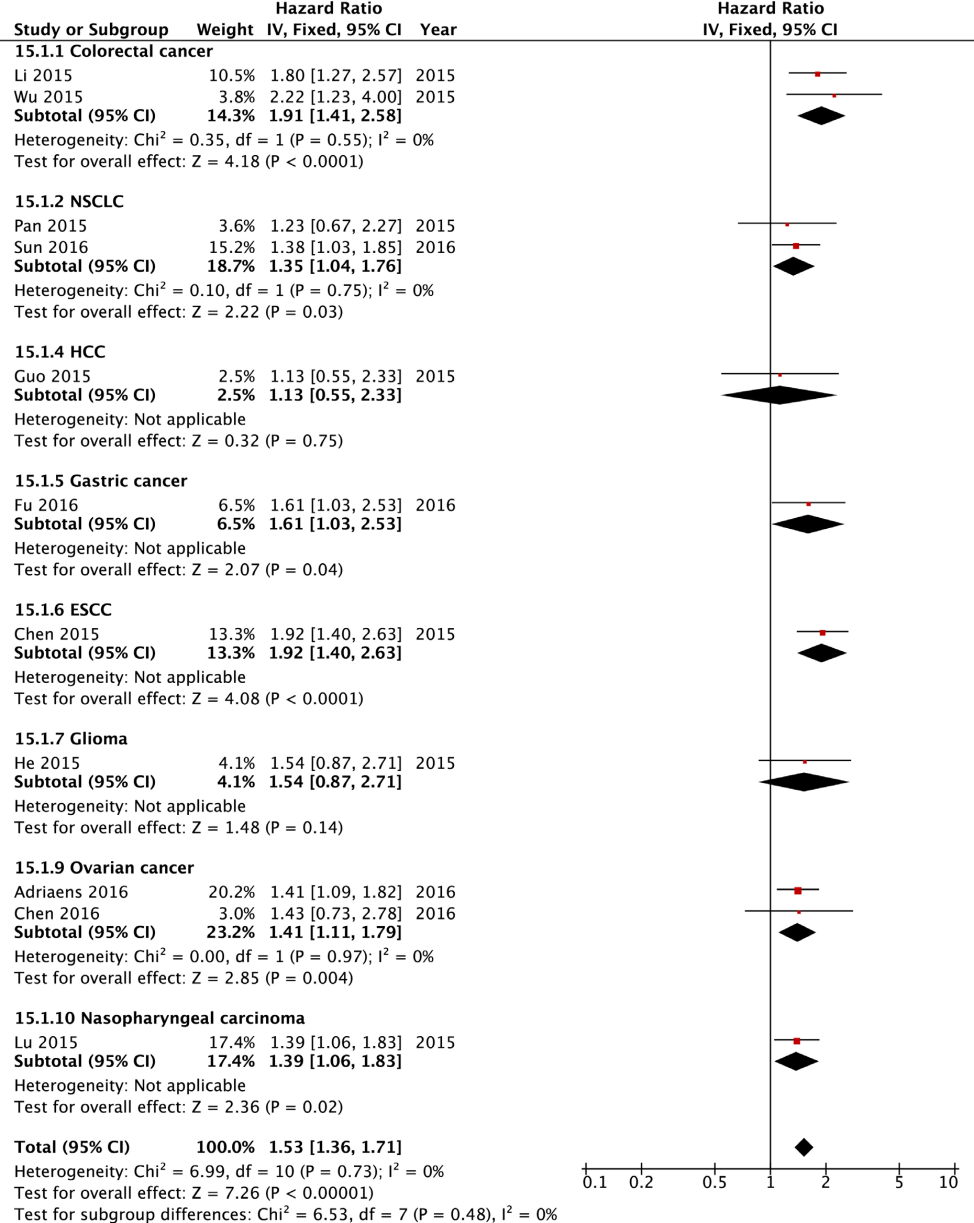
Forest plot of HRs for the association between high NEAT1 expression and OS in cancer patients stratified by different cancer types

We further calculated the pooled HRs for OS based on different type of cancers. As shown in Figure 3, the effects of increased NEAT1 expression on OS was shown in patients with colorectal cancer (HR = 1.91, 95% CI = 1.41–2.58, *P <* 0.001), NSCLC (HR = 1.35, 95% CI = 1.04–1.76, *P* = 0.03), HCC (HR = 1.13, 95% CI = 0.55–2.33, *P* = 0.75), gastric cancer (HR = 1.61, 95% CI = 1.03–2.53, *P* = 0.04), ESCC (HR = 1.92, 95% CI = 1.40–2.63, *P <* 0.001), glioma (HR = 1.48, 95% CI = 0.87–2.71, *P* = 0.14), ovarian cancer (HR = 1.41, 95% CI = 1.11–1.79, *P* = 0.004), and nasopharyngeal carcinoma (HR = 1.39, 95% CI = 1.06–1.83, *P* = 0.02). In addition, we performed subgroup analysis based on cancer types, and similar results were obtained in hepato-gastroenterol cancers (HR = 1.79, 95% CI = 1.48–2.16, *P <* 0.001), NSCLC ((HR = 1.35, 95% CI = 1.04–1.76, *P <* 0.001), ovarian cancers ((HR = 1.41, 95% CI = 1.11–1.79, *P* = 0.004) and other types of grouped cancers (HR = 1.42, 95% CI = 1.11–1.81, *P* = 0.001) (see Table 2 for details and Supplementary Figure S1 for Forest plot).

**Table 2:** Subgroup meta-analysis of pooled HRs for OS

Categories	Studies (*n*)	Number of patients	Fixed-effects model		Heterogeneity	
			HR (95% CI) for OS	*P*-value	I^2^ (%)	*P*_h_
[1] OS	11	1354	1.53 (1.36–1.71)	< 0.001	0	0.73
[2] Cancer type						
1) Hepato-gastroenterol cancers	5	761	1.79 (1.48–2.16)	< 0.001	0	0.65
2) NSCLC	2	221	1.35 (1.04–1.76)	0.03	0	0.75
3) Ovarian cancer	2	207	1.41 (1.11–1.79)	0.004	0	0.97
4) others	2	165	1.42 (1.11–1.81)	0.006	0	0.76
[3] Analysis type						
Multivariate	6	909	1.76 (1.46–2.11)	< 0.001	0	0.72
Survival curves	5	445	1.39 (1.20–1.61)	< 0.001	0	0.99
[4] Sample sizes						
≥ 100	5	844	1.68 (1.35–2.09)	< 0.001	0	0.69
< 100	6	510	1.48 (1.29–1.69)	< 0.001	0	0.58
[5] Cut-off values						
Mean	2	316	1.67 (1.10–2.55)	0.02	46	0.17
Median	4	478	1.47 (1.11–1.96)	0.008	0	0.87
Others	5	560	1.53 (1.34–1.74)	0.001	5	0.38

Further, we also performed subgroup meta-analysis stratified by analysis type, sample size and cut-off value, and similar results were found in regard the effects of increased NEAT1 expression level on OS (See Table 2 for details and see Supplementary Figures S2, S3 and S4 for the Forest plot).

### The association between NEAT1 expression level and disease-free survival (DFS) and progression-free survival (PFS) in cancer patients

In the included study, there was only one study showed the association between NEAT1 expression level and DFS, and one study for association between NEAT1 and PFS. The results of HR showed as high NEAT1 expression versus low NEAT1 expression for DFS in patients with colorectal cancer was 1.80 (95% CI = 1.27–2.55, *P <* 0.001), and for PFS in patient with ovarian cancer was 2.99 (95% CI = 1.21–4.36, *P* = 0.011), which indicated a significantly positive association between high expression level of NEAT1 and poor DFS or poor PFS. However, as one study was performed, the meta-analysis was not performed.

### The association between NEAT1 expression levels and clinicopathological parameters

In order to examine if NEAT1 expression had an association with clinicopathological parameters, we pooled the clinicopathological data for the meta-analysis. As shown in Table 3, the increased NEAT1 expression was significantly associated with larger tumor size (odds ratio (OR) = 1.74, 95% CI = 1.26–2.41, *P <* 0.001, Supplementary Figure S8), lymph node metastasis (OR = 2.29, 95% CI = 1.71–3.06, *P <* 0.001, Supplementary Figure S9), advanced TNM stage (OR = 3.60, 95% CI = 2.27–5.72, *P <* 0.001, Supplementary Figure S10), poor tumor differentiation (OR = 2.16, 95% CI = 1.58–2.93, *P <* 0.001, Supplementary Figure S11), distant metastasis (OR = 3.51, 95% CI = 1.75–7.01, *P <* 0.001, Supplementary Figure S12), and higher invasion depth (OR = 1.94, 95% CI = 1.36–2.75, *P <* 0.001, Supplementary Figure S13). No significant correlation was observed between the increased NEAT1 expression with age, gender, and smoking status (see Table 3 for the details and see Supplementary Figures S5, S6 and S7 for the Forest plots). Because of the insufficient data for other clinicopathological parameters (such as recurrence, lymphatic invasion), the relationship between increased NEAT1 expression level and these clinicopathological parameters were not processed for the meta-analysis.

**Table 3:** Meta-analysis of association between increased NEAT1 expression and clinicopathological parameters

Clinicopathological parameters	Studies (*n*)	Patients (*n*)	OR (95% CI)	*P*-value	Heterogeneity		
					I^2^(%)	P_h_	Model
Age (≥ 55 vs. < 55 years)	7	945	0.95 (0.73–1.25)	0.74	0	0.65	Fixed
Gender (Male vs. Female)	6	796	0.96 (0.72–1.28)	0.77	40	0.14	Fixed
Smoking (Yes vs. No)	2	227	0.96 (0.54–1.71)	0.9	0	0.61	Fixed
Tumor size (≥ 5 cm vs. <5 cm)	5	677	1.74 (1.26–2.41)	< 0.001	0	0.41	Fixed
Lymph node metastasis (Yes vs. No)	6	806	2.29 (1.71–3.06)	< 0.001	18	0.3	Fixed
TNM stage (III–IV vs. I–II)	7	945	3.60 (2.27–5.72)	< 0.001	58	0.03	Random
Tumor differentiation (Poor vs. Moderate/Well)	5	334	2.16 (1.58–2.93)	< 0.001	0	0.77	Fixed
Distant metastasis (Yes vs. No)	5	755	3.51 (1.75–7.01)	< 0.001	61	0.04	Random
Invasion depth (T3–T4 vs. T1–T2)	3	276	1.94 (1.36–2.75)	< 0.001	0	0.49	Fixed

### Sensitivity analysis

For the meta-analysis of the association between NEAT1 expression level and OS, the sensitivity analysis was performed by removing each study in turn from the pooled analysis. This analysis functions to examine the impact of the removed study on the overall HRs. In the present study, removing any of the included studies had no significant influence on the results, which suggests the robustness of the results.

### Analysis of publication bias

**Figure 4:**
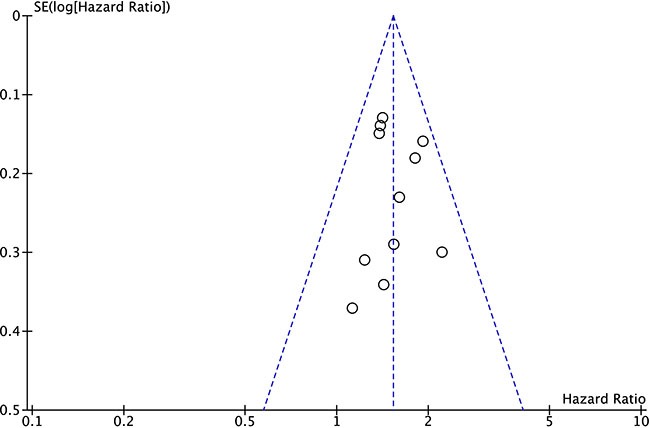
Funnel plot analysis for potential publication bias among included eligible studies

In order to assess whether publication bias was existed in the included studies regarding the association between NEAT1 expression and OS, we performed the funnel plot analysis and applied the trim and filled methods (Figure 4), and our results demonstrated that on obvious publication bias was presented in the included studies for meta-analysis.

## DISCUSSION

NEAT1 is a ~3.2 kb novel nuclear long non-coding RNA, located in chromosome 11q13.1 [15]. Recent studies identified NEAT1 as a crucial architectural component of a paraspeckle structure, and NEAT1 has been shown to regulate numerous biological processes including cellular differentiation and stress response through paraspeckles pathway [15–17]. In the cancer studies, NEAT1 was found to be up-regulated in various types of cancer tissues and cancer cell lines, and NEAT1 was a key mediator in cancer progression by the regulation of cell apoptosis, cell proliferation as well as cell cycle [18]. In this regard, NEAT1 has been suggest as a potential diagnostic marker and may represent a novel target for the treatment of cancers. Apart from this, NEAT1 was also found to contribute to the chemo-/radio-resistance in ovarian cancer, lung cancer and nasopharyngeal carcinoma, which implicated that NEAT1 could be a potential biomarker for chemo-sensitivity.

A lot of efforts have been made to understand the functional role of NEAT1 in cancer progression, but the underlying molecular mechanisms of NEAT1 involved cancer progression are largely unknown. An important recent study demonstrated that NEAT1-containing paraspeckles could be induced by p53 which in turn modulates the replication stress and chemosensitivity in cancer cells [19]. NEAT1 can function to be oncogenic by sponging the tumor-suppressive microRNAs. Study from Zhen et al., 2016 showed that NEAT1 promoted glioma pathogenesis by interacting with miR-449b-5p/c-Met axis [20]. NEAT1 was also found to promote laryngeal squamous cell cancer through regulating miR-107/CDK6 pathway [21]. In the breast cancer, NEAT1 is required for survival of breast cancer cells via targeting miR-548 [22]. In terms of chemo-resistance, NEAT1 was found to up-regulated EGCG-induced CTR1 to enhance cisplatin sensitivity in lung cancer cells [23]. These results may suggest that targeting NEAT1 may be beneficial for the treatment of human cancers. However, the role of NEAT1 in other non-studied types of cancer may be further investigated to confirm the role of NEAT1.

In the present study, the meta-analysis results implied that high NEAT1 expression was significantly associated with poor prognosis in patients with different types of cancers. The pooled HRs results showed that increased NEAT1 expression was positively associated with a shorter OS in patients with different types of cancers, which suggests the prognostic role of NEAT1 in predicting OS in cancer patients. Further subgroup analysis for OS showed that increased NEAT1 in cancer patients may be a reliable prognostic factor for hepato-gastroenterol cancers. Apart from the included studies for solid tumors, NEAT1 was also found to have regulatory role in leukemia [24], however, the lack of relevant clinical data precluded it from meta-analysis in the present study. Apart from the function role NEAT1 in caner, NEAT1 also had other functional roles. For instances, NEAT1 was found to contribute to the pathogenesis of lupus [25]; altered expression of NEAT1 was also found in the Huntington's disease [26]; NEAT1 is required for mammary gland development and lactation [27], which suggests the diverse functional roles of NEAT1.

In the included studies from present study, only one study from Li et al., 2015 reported the association between increased NEAT1 expression and DFS in colorectal cancer [11], and only one study from Adriaens et al., 2016 reported the association between increased NEAT1 expression and PFS in ovarian cancer [19], and thus meta-analysis was not performed in these two studies. However, these studies suggested the prognostic role of NEAT1 for DFS as well as PFS in these cancer patients. The meta-analysis for the association between increased NEAT1 expression and clinicopathological parameters was also analyzed in this study, and our results showed that increased NEAT1 expression was significantly associated with larger tumor size, lymph node metastasis, advanced TNM stage, poor tumor differentiation, distant metastasis, and higher invasion depth, which may suggest that increased NEAT1 may be associated with advanced features of cancer.

However, there are still some limitations in our meta-analysis from this study. For example, the total sample size was relatively small, and most of the patients included in the meta-analysis were from China. In addition, publication bias may exist, despite the fact that no significant publication bias was observed based on stable results revealed in sensitivity analysis as well as funnel plot analysis. Finally, the cut-off values definition for high NEAT1 expression was not consistent among the included. Therefore, larger-size, multi-center and higher-quality studies with unified criteria for determining NEAT1 expression are necessary to solidify the results in this study.

In conclusion, the meta-analysis results suggest the prognostic role NEAT1 in prognosis in the patients with different types of cancer. However, due to several limitations of the included studies, larger-sample size, multi-center and higher-quality studies with consistent criteria for defining high NEAT1 expression level and low NEAT1 expression level may be required to further confirm the current findings in this study.

## MATERIALS AND METHODS

### Literature search

To retrieve potentially eligible studies, comprehensive literature search was performed in the following databases: PubMed, Web of Science, Embase, and CNKI, and the cut-off date was defined as September 30, 2016. The keywords for the search in these databases included: “nuclear enriched abundant transcript 1”, “NEAT1”, “long non-coding RNA NEAT1”, “lncRNA NEAT1”, “cancer”, “tumor”, “carcinoma”, “neoplasm”, and other eligible literatures were also manually evaluated from the references lists.

### Inclusion and exclusion criteria

Inclusion criteria for the eligible studies included: (a) associations of NEAT1 expression levels with prognosis or clinicopathological features were described, (b) the role of NEAT1 in human cancer development was examined, (c) patients were categorized into two groups based on high and low expression levels of NEAT1, (d) the expression levels of NEAT1 in the cancer patients were determined by qRT-PCR. Exclusion criteria for the articles included: (a) studies without presenting data with relevant values, (b) duplicated publications, (c) letters, reviews, case reports and expert opinions.

### Data extraction and quality assessment

The data and information from all included eligible studies were independently evaluated by two investigators (ZL and YL). The following information were extracted from each eligible study: the name of first author, year of publication, cancer type, total number of patients from each eligible study, TNM stage, follow-up period, outcome measures, method for detecting NEAT1 expression, determination method, hazard ratio and its corresponding 95% confident interval, the clinicopathological parameters from each eligible study. For the eligible studies that provided both the univariate and multivariate analysis, the multivariate values were chosen as the multivariate values had higher precision on interpreting confounding factors. In the eligible studies only reporting Kaplan-Meier curves, the software, Enguage Digitizer (Version 4.1) was used to extract the survival data. In the situation of a disagreement, a consensus was reached by a third investigator (RX). The quality of all the included studies were assessed by The Newcastle-Ottawa Scale (NOS) method. The NOS scores ranged from 0 to 9, and a study with an NOS score more than 6 was regarded as high quality.

### Statistical methods

The meta-analysis was performed with RevMan 5.3 software and Stata SE12.0. The heterogeneity between studies was determined by the Chi square-based *Q* test and I^2^ statistics. *P <* 0.05 for the *Q* test (P_h_) and I^2^ > 50% were considered to be significantly heterogeneous. The fixed effects model was applied in the studies with no obvious heterogeneity (P_h_ > 0.05, I^2^ < 50%); the random effects model was applied in the studies with obvious heterogeneity (P_h_ ≤ 0.05, I^2^ ≥ 50%). The sensitivity analysis was also carried out to assess the stability of the results. A *P* values less than 0.05 was considered to be statistically significant.

## SUPPLEMENTARY MATERIALS FIGURES


