# Supplementary material for: Long non-coding RNA NEAT1 overexpression is associated with poor prognosis in cancer patients: a systematic review and meta-analysis
Source: Oncotarget. 2016 Dec 1;8(2):2672–80. doi: 10.18632/oncotarget.13737 (PMC5356832; doi:10.18632/oncotarget.13737)
Supplement: Supplementary file 1 [file oncotarget-08-2672-s001.pdf]

Long non-coding RNA NEAT1 overexpression is associated with poor prognosis in cancer patients: a systematic review and meta-analysis

Supplementary Materials

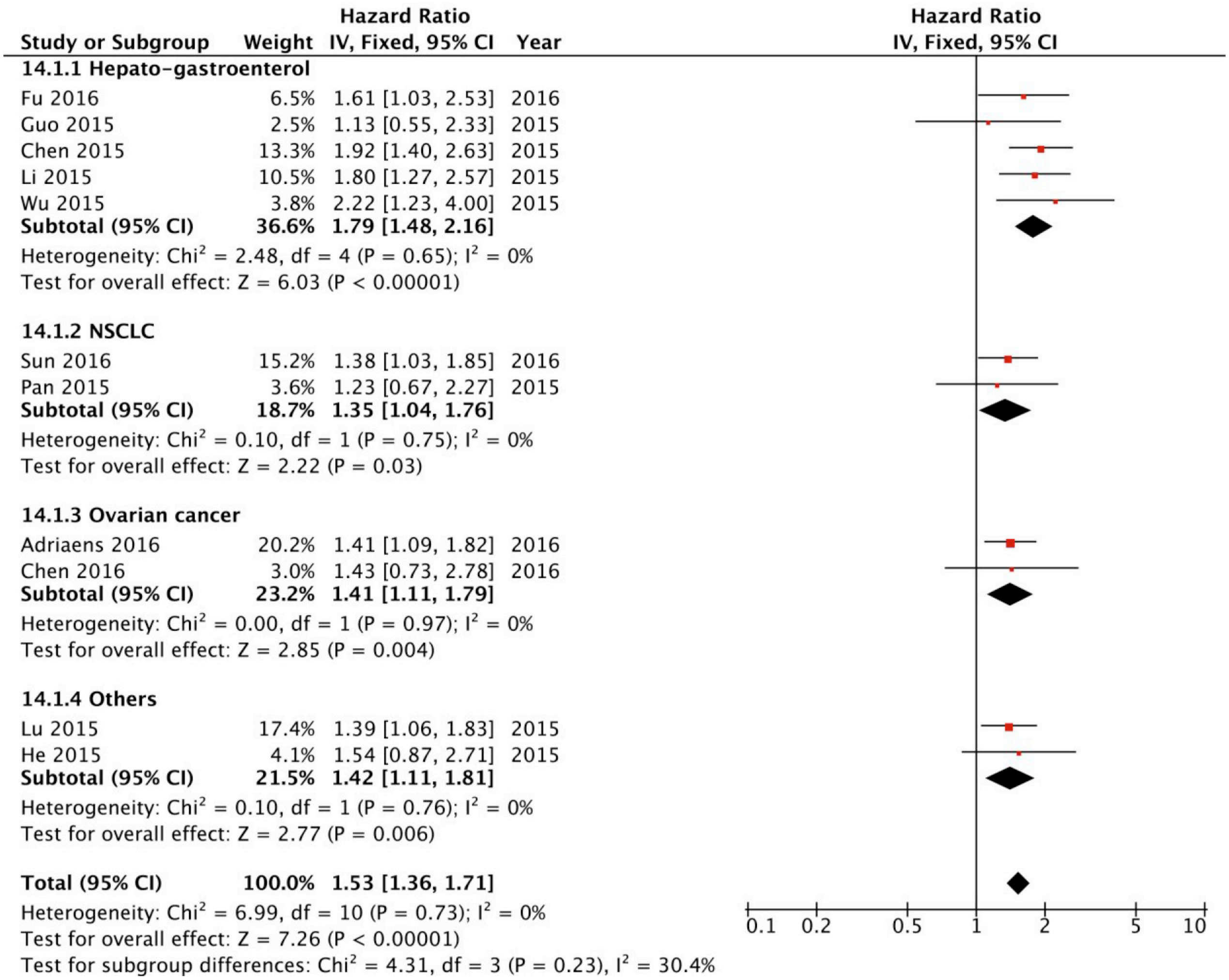

Supplementary Figure S1: Forest plot of HRs for the association between increased NEAT1 and OS subgrouped by cancer types.

Study or Subgroup	Weight	Hazard Ratio IV, Fixed, 95% CI	Year
**16.1.1 Multivariate analysis**			
Chen 2016	3.0%	1.43 [0.73, 2.78]	2016
Fu 2016	6.5%	1.61 [1.03, 2.53]	2016
Chen 2015	13.3%	1.92 [1.40, 2.63]	2015
Wu 2015	3.8%	2.22 [1.23, 4.00]	2015
Guo 2015	2.5%	1.13 [0.55, 2.33]	2015
Li 2015	10.5%	1.80 [1.27, 2.57]	2015
**Subtotal (95% CI)**	**39.6%**	**1.76 [1.47, 2.11]**	
Heterogeneity: $Chi^2 = 2.89$, df = 5 (P = 0.72); $I^2 = 0\%$			
Test for overall effect: Z = 6.09 (P < 0.00001)			
**16.1.2 Survival curves**			
Adriaens 2016	20.2%	1.41 [1.09, 1.82]	2016
Sun 2016	15.2%	1.38 [1.03, 1.85]	2016
Pan 2015	3.6%	1.23 [0.67, 2.27]	2015
He 2015	4.1%	1.54 [0.87, 2.71]	2015
Lu 2015	17.4%	1.39 [1.06, 1.83]	2015
**Subtotal (95% CI)**	**60.4%**	**1.39 [1.20, 1.61]**	
Heterogeneity: $Chi^2 = 0.28$, df = 4 (P = 0.99); $I^2 = 0\%$			
Test for overall effect: Z = 4.41 (P < 0.0001)			
**Total (95% CI)**	**100.0%**	**1.53 [1.36, 1.71]**	
Heterogeneity: $Chi^2 = 6.99$, df = 10 (P = 0.73); $I^2 = 0\%$			
Test for overall effect: Z = 7.26 (P < 0.00001)			
Test for subgroup differences: $Chi^2 = 3.82$, df = 1 (P = 0.05), $I^2 = 73.8\%$			

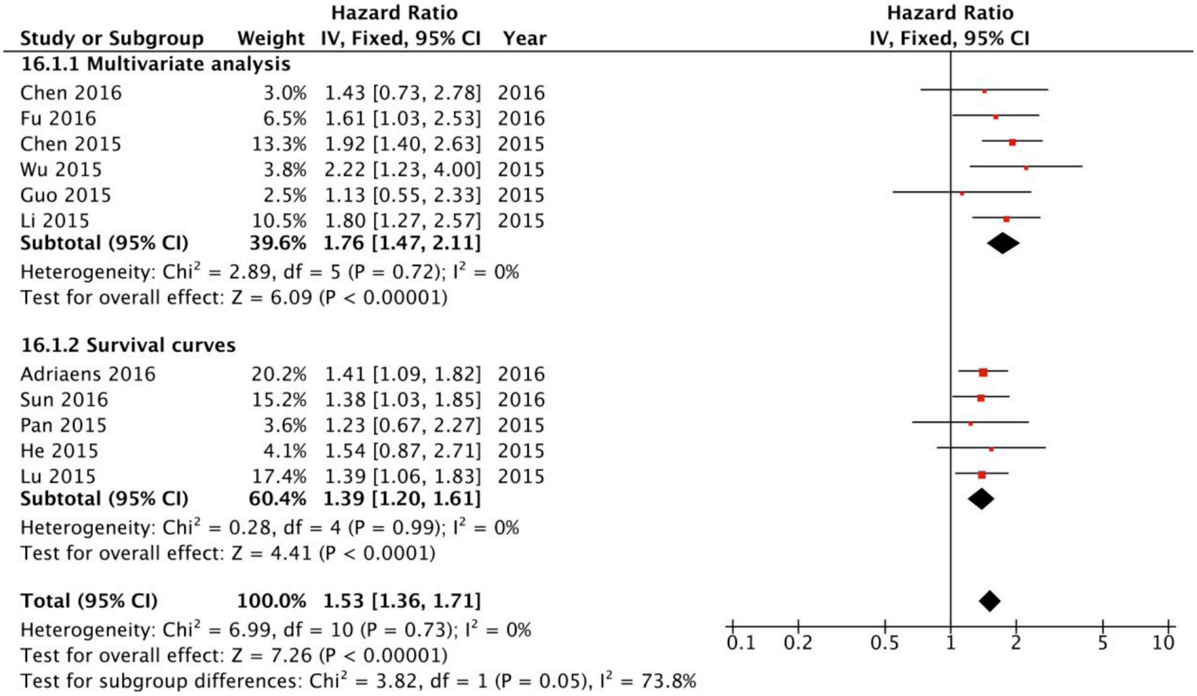

Supplementary Figure S2: Forest plot of HRs for the association between increased NEAT1 and OS in cancer patients stratified by analysis methods.

Study or Subgroup	Weight	Hazard Ratio IV, Fixed, 95% CI	Year
**17.1.1 sample size (>100)**			
Chen 2016	3.0%	1.43 [0.73, 2.78]	2016
Fu 2016	6.5%	1.61 [1.03, 2.53]	2016
Wu 2015	3.8%	2.22 [1.23, 4.00]	2015
Pan 2015	3.6%	1.23 [0.67, 2.27]	2015
Li 2015	10.5%	1.80 [1.27, 2.57]	2015
**Subtotal (95% CI)**	**27.3%**	**1.68 [1.35, 2.09]**	
Heterogeneity: $Chi^2 = 2.27$, df = 4 (P = 0.69); $I^2 = 0\%$			
Test for overall effect: Z = 4.63 (P < 0.00001)			
**17.1.2 sample size (<100)**			
Adriaens 2016	20.2%	1.41 [1.09, 1.82]	2016
Sun 2016	15.2%	1.38 [1.03, 1.85]	2016
Chen 2015	13.3%	1.92 [1.40, 2.63]	2015
Guo 2015	2.5%	1.13 [0.55, 2.33]	2015
He 2015	4.1%	1.54 [0.87, 2.71]	2015
Lu 2015	17.4%	1.39 [1.06, 1.83]	2015
**Subtotal (95% CI)**	**72.7%**	**1.48 [1.29, 1.69]**	
Heterogeneity: $Chi^2 = 3.76$, df = 5 (P = 0.58); $I^2 = 0\%$			
Test for overall effect: Z = 5.68 (P < 0.00001)			
**Total (95% CI)**	**100.0%**	**1.53 [1.36, 1.71]**	
Heterogeneity: $Chi^2 = 6.99$, df = 10 (P = 0.73); $I^2 = 0\%$			
Test for overall effect: Z = 7.26 (P < 0.00001)			
Test for subgroup differences: $Chi^2 = 0.96$, df = 1 (P = 0.33), $I^2 = 0\%$			

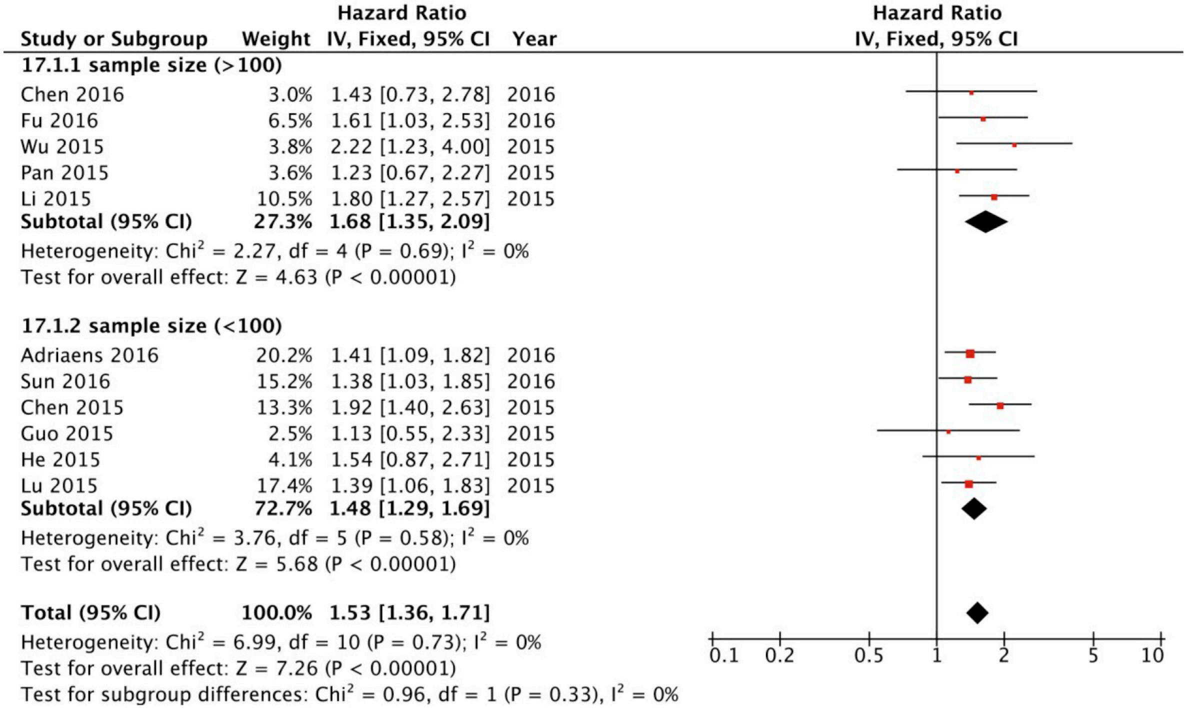

Supplementary Figure S3: Forest plot of HRs for the association between increased NEAT1 and OS in cancer patients stratified by sample size.

Study or Subgroup	Weight	Hazard Ratio IV, Fixed, 95% CI	Year
**18.1.1 Mean**			
Wu 2015	3.8%	2.22 [1.23, 4.00]	2015
Pan 2015	3.6%	1.23 [0.67, 2.27]	2015
**Subtotal (95% CI)**	**7.3%**	**1.67 [1.10, 2.55]**	
Heterogeneity: $Chi^2 = 1.86$, df = 1 (P = 0.17); $I^2 = 46\%$			
Test for overall effect: Z = 2.38 (P = 0.02)			
**18.1.2 Median**			
Fu 2016	6.5%	1.61 [1.03, 2.53]	2016
Chen 2016	3.0%	1.43 [0.73, 2.78]	2016
He 2015	4.1%	1.54 [0.87, 2.71]	2015
Guo 2015	2.5%	1.13 [0.55, 2.33]	2015
**Subtotal (95% CI)**	**16.0%**	**1.47 [1.11, 1.96]**	
Heterogeneity: $Chi^2 = 0.70$, df = 3 (P = 0.87); $I^2 = 0\%$			
Test for overall effect: Z = 2.65 (P = 0.008)			
**18.1.3 Others**			
Adriaens 2016	20.2%	1.41 [1.09, 1.82]	2016
Sun 2016	15.2%	1.38 [1.03, 1.85]	2016
Chen 2015	13.3%	1.92 [1.40, 2.63]	2015
Li 2015	10.5%	1.80 [1.27, 2.57]	2015
Lu 2015	17.4%	1.39 [1.06, 1.83]	2015
**Subtotal (95% CI)**	**76.7%**	**1.53 [1.34, 1.74]**	
Heterogeneity: $Chi^2 = 4.19$, df = 4 (P = 0.38); $I^2 = 5\%$			
Test for overall effect: Z = 6.35 (P < 0.00001)			
**Total (95% CI)**	**100.0%**	**1.53 [1.36, 1.71]**	
Heterogeneity: $Chi^2 = 6.99$, df = 10 (P = 0.73); $I^2 = 0\%$			
Test for overall effect: Z = 7.26 (P < 0.00001)			
Test for subgroup differences: $Chi^2 = 0.24$, df = 2 (P = 0.89), $I^2 = 0\%$			

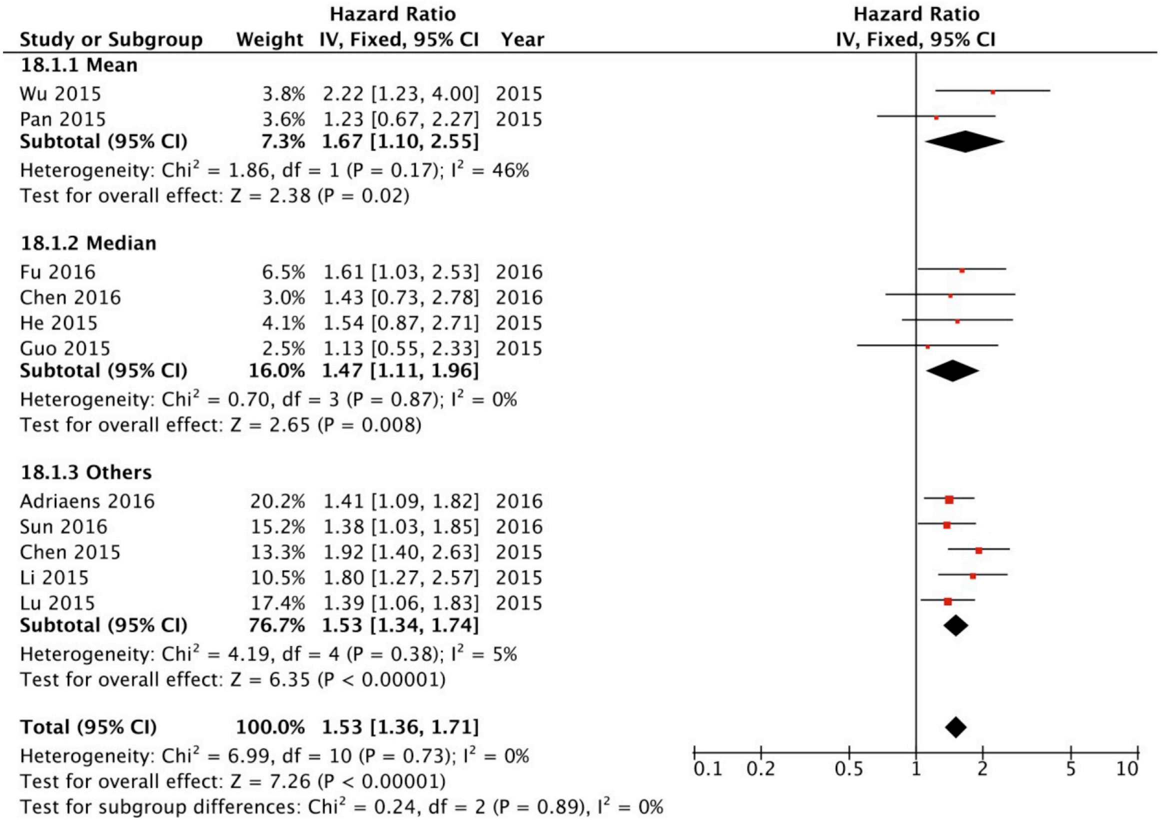

Supplementary Figure S4: Forest plot of HRs for the association between increased NEAT1 and OS in cancer patients stratified by cut-off value.

Study or Subgroup	Age≥55 years Events	Total	Age<55 years Events	Total	Weight	Odds Ratio M-H, Fixed, 95% CI	Year
Chen 2016	39	83	35	66	18.4%	0.79 [0.41, 1.50]	2016
Fu 2016	45	86	25	54	13.0%	1.27 [0.64, 2.52]	2016
Sun 2016	26	34	41	62	6.1%	1.66 [0.64, 4.31]	2016
Chen 2015	25	49	29	47	12.9%	0.65 [0.29, 1.46]	2015
Lu 2015	31	67	35	64	17.1%	0.71 [0.36, 1.42]	2015
Li 2015	45	95	65	144	24.2%	1.09 [0.65, 1.84]	2015
He 2015	35	71	12	23	8.2%	0.89 [0.35, 2.28]	2015
**Total (95% CI)**		**485**		**460**	**100.0%**	**0.96 [0.73, 1.25]**	
Total events	246		242				
Heterogeneity: $Chi^2 = 4.21$, df = 6 (P = 0.65); $I^2 = 0\%$							
Test for overall effect: Z = 0.34 (P = 0.74)							

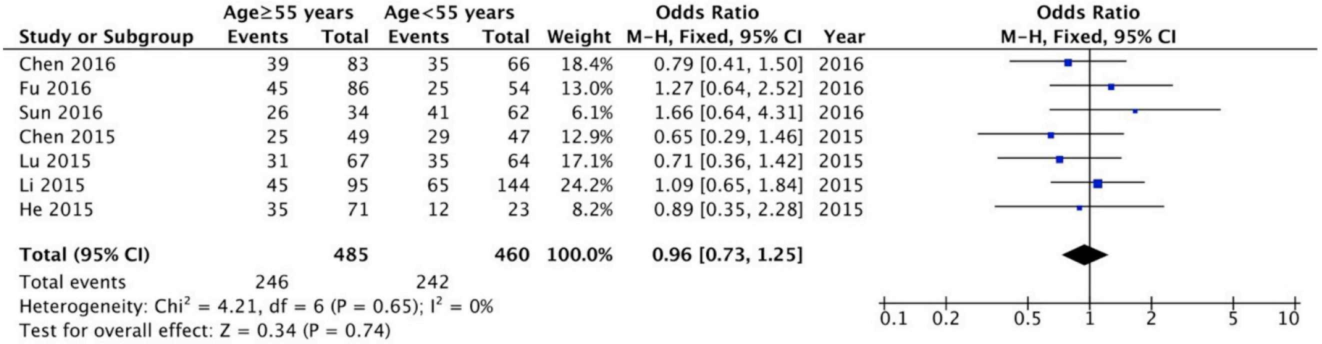

Supplementary Figure S5: Forest plot of HRs for the association between increased NEAT1 and age in cancer patients.

Study or Subgroup	Female Events	Female Total	Male Events	Male Total	Weight	Odds Ratio M-H, Fixed, 95% CI	Year
Fu 2016	23	55	47	85	23.1%	0.58 [0.29, 1.15]	2016
Sun 2016	27	36	40	60	8.1%	1.50 [0.59, 3.79]	2016
Chen 2015	17	24	37	72	5.8%	2.30 [0.85, 6.21]	2015
Li 2015	44	98	66	141	32.0%	0.93 [0.55, 1.55]	2015
Lu 2015	32	71	34	60	21.7%	0.63 [0.31, 1.25]	2015
He 2015	18	32	29	62	9.3%	1.46 [0.62, 3.45]	2015
**Total (95% CI)**		**316**		**480**	**100.0%**	**0.96 [0.72, 1.28]**	
Total events	161		253				

Heterogeneity: $Chi^2 = 8.30$, df = 5 (P = 0.14); $I^2 = 40\%$

Test for overall effect: Z = 0.30 (P = 0.77)

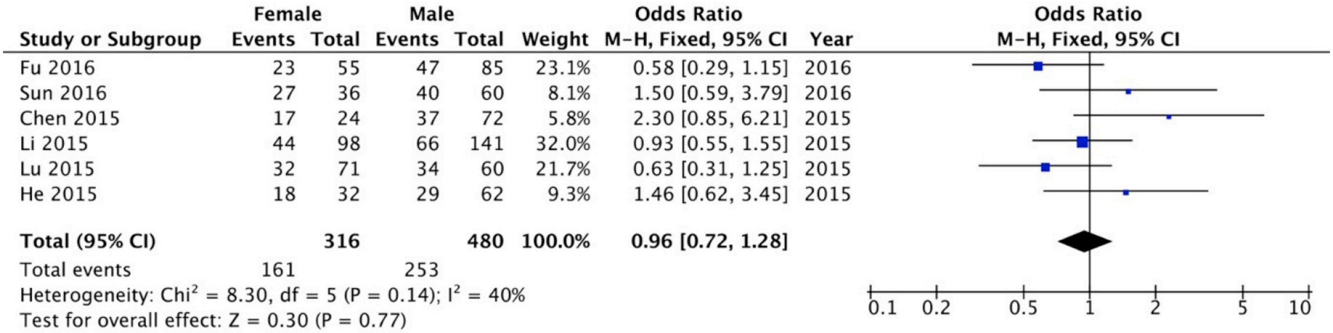

Supplementary Figure S6: Forest plot of HRs for the association between increased NEAT1 and gender in cancer patients.

Study or Subgroup	Yes Events	Yes Total	No Events	No Total	Weight	Odds Ratio M-H, Fixed, 95% CI	Year
Sun 2016	50	73	17	23	34.3%	0.77 [0.27, 2.20]	2016
Lu 2015	28	60	32	71	65.7%	1.07 [0.54, 2.13]	2015
**Total (95% CI)**		**133**		**94**	**100.0%**	**0.96 [0.54, 1.71]**	
Total events	78		49				

Heterogeneity: $Chi^2 = 0.26$, df = 1 (P = 0.61); $I^2 = 0\%$

Test for overall effect: Z = 0.13 (P = 0.90)

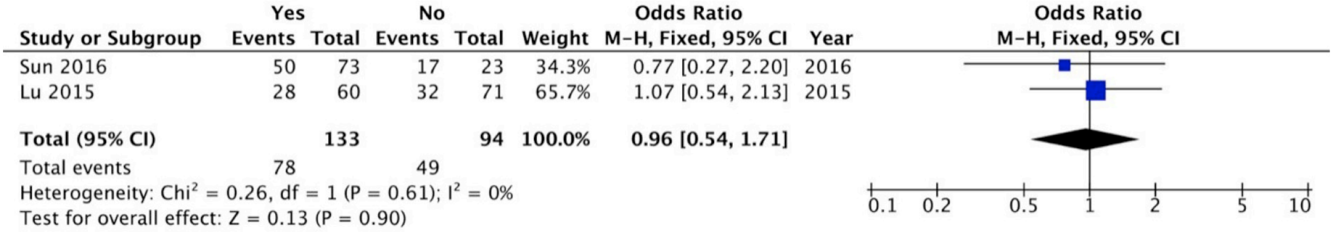

Supplementary Figure S7: Forest plot of HRs for the association between increased NEAT1 and smoking status in cancer patients.

Study or Subgroup	Tumor size ≥ 5 cm Events	Tumor size ≥ 5 cm Total	Tumor size < 5 cm Events	Tumor size < 5 cm Total	Weight	Odds Ratio M-H, Fixed, 95% CI	Year
Chen 2016	33	64	41	85	30.8%	1.14 [0.60, 2.19]	2016
Sun 2016	46	55	27	41	9.1%	2.65 [1.01, 6.94]	2016
Chen 2015	42	66	12	30	10.8%	2.63 [1.08, 6.37]	2015
Li 2015	78	157	32	82	38.1%	1.54 [0.90, 2.66]	2015
He 2015	37	67	10	30	11.2%	2.47 [1.00, 6.06]	2015
**Total (95% CI)**		**409**		**268**	**100.0%**	**1.74 [1.26, 2.41]**	
Total events	236		122				

Heterogeneity: $Chi^2 = 3.94$, df = 4 (P = 0.41); $I^2 = 0\%$

Test for overall effect: Z = 3.35 (P = 0.0008)

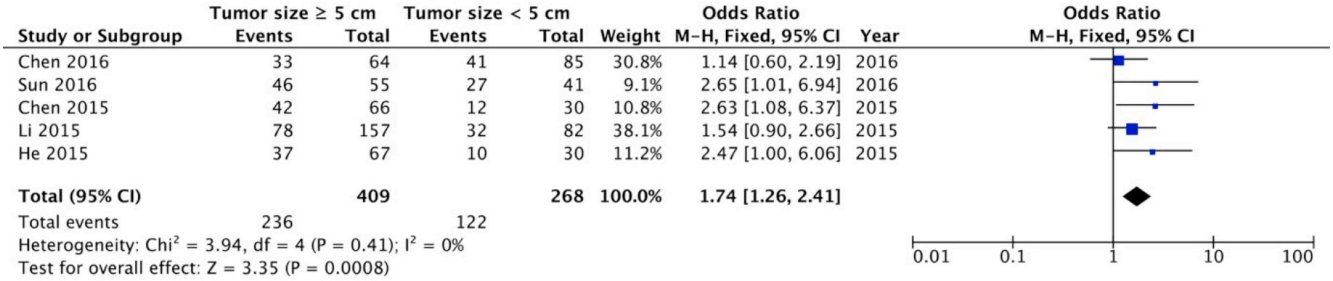

Supplementary Figure S8: Forest plot of HRs for the association between increased NEAT1 and tumor size in cancer patients.

Study or Subgroup	Yes Events	Yes Total	No Events	No Total	Weight	Odds Ratio M-H, Fixed, 95% CI	Year	Odds Ratio M-H, Fixed, 95% CI
Fu 2016	50	80	20	70	13.1%	4.17 [2.09, 8.29]	2016	
Sun 2016	32	39	35	57	8.4%	2.87 [1.08, 7.63]	2016	
He 2015	31	50	16	44	10.6%	2.86 [1.23, 6.61]	2015	
Li 2015	76	147	34	92	33.1%	1.83 [1.07, 3.11]	2015	
Lu 2015	39	70	29	61	22.5%	1.39 [0.70, 2.77]	2015	
Chen 2015	32	50	20	46	12.3%	2.31 [1.02, 5.25]	2015	
**Total (95% CI)**		**436**		**370**	**100.0%**	**2.29 [1.71, 3.06]**		
Total events	260		154					

Heterogeneity: $Chi^2 = 6.10$, df = 5 (P = 0.30); $I^2 = 18\%$
Test for overall effect: Z = 5.60 (P < 0.00001)

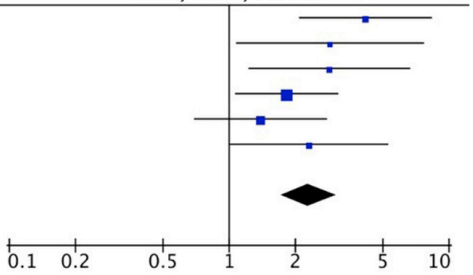

Supplementary Figure S9: Forest plot of HRs for the association between increased NEAT1 and lymph node metastasis in cancer patients.

Study or Subgroup	TNM stage (I–II) Events	TNM stage (I–II) Total	TNM stage (III–IV) Events	TNM stage (III–IV) Total	Weight	Odds Ratio M-H, Random, 95% CI	Year	Odds Ratio M-H, Random, 95% CI
Fu 2016	55	77	15	63	15.1%	8.00 [3.73, 17.14]	2016	
Chen 2016	56	96	18	53	16.1%	2.72 [1.35, 5.47]	2016	
Sun 2016	54	68	13	28	12.3%	4.45 [1.73, 11.48]	2016	
Lu 2015	44	61	36	70	15.6%	2.44 [1.18, 5.07]	2015	
Li 2015	76	147	34	92	19.0%	1.83 [1.07, 3.11]	2015	
He 2015	44	71	3	23	8.4%	10.86 [2.95, 40.05]	2015	
Chen 2015	41	61	13	35	13.4%	3.47 [1.45, 8.28]	2015	
**Total (95% CI)**		**581**		**364**	**100.0%**	**3.60 [2.27, 5.72]**		
Total events	370		132					

Heterogeneity: $Tau^2 = 0.22$; $Chi^2 = 14.43$, df = 6 (P = 0.03); $I^2 = 58\%$
Test for overall effect: Z = 5.42 (P < 0.00001)

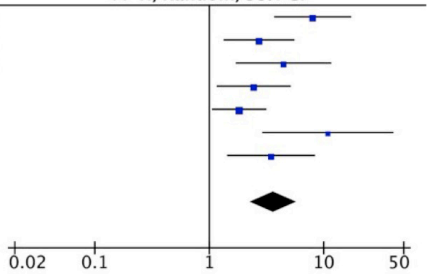

Supplementary Figure S10: Forest plot of HRs for the association between increased NEAT1 and TNM stage in cancer patients.

Study or Subgroup	Poor Events	Poor Total	Well/Moderate Events	Well/Moderate Total	Weight	Odds Ratio M-H, Fixed, 95% CI	Year	Odds Ratio M-H, Fixed, 95% CI
Sun 2016	33	44	34	52	14.2%	1.59 [0.65, 3.87]	2016	
Chen 2016	52	89	22	60	20.0%	2.43 [1.24, 4.76]	2016	
Fu 2016	43	74	27	66	21.9%	2.00 [1.02, 3.93]	2016	
Li 2015	49	95	41	144	28.8%	2.68 [1.56, 4.60]	2015	
Chen 2015	15	37	18	59	15.1%	1.55 [0.66, 3.67]	2015	
**Total (95% CI)**		**339**		**381**	**100.0%**	**2.16 [1.58, 2.93]**		
Total events	192		142					

Heterogeneity: $Chi^2 = 1.79$, df = 4 (P = 0.77); $I^2 = 0\%$
Test for overall effect: Z = 4.87 (P < 0.00001)

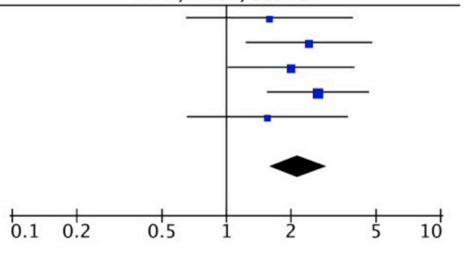

Supplementary Figure S11: Forest plot of HRs for the association between increased NEAT1 and tumor differentiation in cancer patients.

Study or Subgroup	Yes Events	Yes Total	No Events	No Total	Weight	Odds Ratio M-H, Random, 95% CI	Year
Chen 2016	45	56	29	93	24.3%	9.03 [4.09, 19.93]	2016
Fu 2016	8	8	62	132	5.0%	19.18 [1.08, 339.03]	2016
Li 2015	20	31	90	208	24.5%	2.38 [1.09, 5.23]	2015
Chen 2015	16	23	38	73	20.5%	2.11 [0.77, 5.72]	2015
Lu 2015	48	67	34	64	25.7%	2.23 [1.08, 4.59]	2015
**Total (95% CI)**		**185**		**570**	**100.0%**	**3.51 [1.75, 7.01]**	
Total events	137		253				

Heterogeneity: $Tau^2 = 0.35$; $Chi^2 = 10.28$, df = 4 (P = 0.04); $I^2 = 61\%$

Test for overall effect: Z = 3.55 (P = 0.0004)

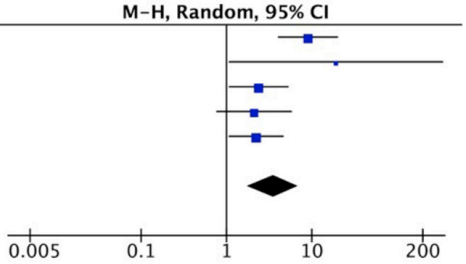

Supplementary Figure S12: Forest plot of HRs for the association between increased NEAT1 and distant metastasis in cancer patients.

Study or Subgroup	T3-T4 Events	T3-T4 Total	T1-T2 Events	T1-T2 Total	Weight	Odds Ratio M-H, Fixed, 95% CI	Year
Li 2015	76	147	35	92	46.4%	1.74 [1.03, 2.96]	2015
Lu 2015	37	64	31	67	28.5%	1.59 [0.80, 3.17]	2015
Fu 2016	55	77	42	87	25.1%	2.68 [1.40, 5.13]	2016
**Total (95% CI)**		**288**		**246**	**100.0%**	**1.94 [1.36, 2.75]**	
Total events	168		108				

Heterogeneity: $Chi^2 = 1.42$, df = 2 (P = 0.49); $I^2 = 0\%$

Test for overall effect: Z = 3.68 (P = 0.0002)

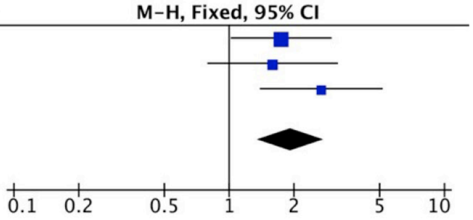

Supplementary Figure S13: Forest plot of HRs for the association between increased NEAT1 and invasion depth in cancer patients.
